# Sedentary behavior accelerates biological aging mediated by body mass index in adults

**DOI:** 10.1038/s41598-025-06325-x

**Published:** 2025-07-01

**Authors:** Jinhang Che, Jie Huai, Lihong Luo, Yunxiang Tang, Tao Zeng

**Affiliations:** 1 Department of Electrocardiographic Diagnosis, The Fourth People’s Hospital of Chengdu, Chengdu, China; 2 Department of Ultrasonography, The Fourth People’s Hospital of Chengdu, No. 8, Huli West Lane, Yingmen, Jinniu District, Chengdu, 610000 China

**Keywords:** Sedentary behavior, Phenotypic age, Body mass index, Sitting time, Risk factors, Public health

## Abstract

Sedentary behavior is widely recognized as a detriment to health. Limited conclusions have been drawn about the relationship between sitting time and biomarkers-measured aging. 12,504 eligible adults were included from the National Health and Nutrition Examination Survey (NHANES) 2007 to 2016. Weighted logistic regression, subgroup analysis, and restricted cubic spline regression were conducted to investigate the association and dose-response relationship between sitting time and phenotypic age acceleration (PhenoAgeAccel). The mediating effect of body mass index (BMI) on this correlation was revealed by mediation analysis. After adjusting for multiple covariates, longer sitting time (4–6 h: OR 1.30, 95%CI 1.06–1.58, *p* = 0.013; 6–8 h: OR 1.25, 95%CI 1.01–1.55, *p* = 0.038; ≥8 h: OR 1.58, 95%CI 1.33–1.88, *p* < 0.001) significantly had higher risk of aging comparing to the reference (< 4 h). The dose-response relationship exhibited an approximately linear dependence. Additionally, BMI partially mediated the association between sitting time and PhenoAgeAccel by a 21.0% proportion. Our study revealed a strong, significant, independent, linear relationship between sitting time and phenotypic age. BMI served as a mediator of the correlation between sitting time and PhenoAgeAccel.

## Introduction

Sedentary behavior is commonly defined as any awake activity performed while sitting, reclining, or lying down, with an energy expenditure ≤ 1.5 metabolic equivalents^1^. Because of the widespread use of electronic devices, changes in transportation patterns, growth of sedentary occupations, sedentary behavior has become common in modern society^2^. Recent studies conclude that sedentary behavior is a risk factor for various diseases, such as metabolic syndrome, cardiovascular disease, Parkinson’s disease, increase of body fat, and decrease of bone density^2–5^. Therefore, sedentary behavior is regarded as a modifiable risk factor for several diseases. As recommended by the World Health Organization (WHO) 2020 Global Guidelines on Physical Activity and Sedentary Behavior, adults should limit their sedentary time and replace it with physical activities.

Aging represents the deterioration of biological functions and dysregulation of multiple physiological systems^6^. Unquestionably, aging is an inevitable process, while the rate of aging is heterogeneous. Accelerated aging increases the susceptibility to death and diseases^7^. At the same time, it’s possible to avoid or postpone the onset of age-related diseases by slowing the rate of aging^8^. Phenotypic age (PhenoAge) serves as an emerging indicator of an individual’s biological age^9^ and has been shown to outperform in capturing health risk relative to telomere length^10^.

Recently, limited studies conclude that prolonged sedentary behavior may accelerate the biological aging process^11,12^. Sedentary behavior is related to a higher BMI, which has been shown to be linked to a biological aging^13^. Unlike molecular markers, BMI is routinely measured in clinical practice and effectively modified through lifestyle changes. Considering the limitations of previous studies, we aim to examine the association of sitting time and PhenoAge among adults in the U.S. population and we hypothesize that sedentary activity accelerates biological aging and BMI plays a mediating role.

## Method

### Data source

The National Health and Nutrition Examination Survey (NHANES) dataset is a public, cross-sectional, countrywide, two-year cycled survey which collects demographic, socioeconomic, health, and nutritional information from the US population. Conducted by the National Center for Health Statistics (NCHS) of the Centers for Disease Control and Prevention (CDC), NHANES employs a complex, multistage, stratified, clustered probabilistic design and written informed consent are obtained from all participants^14^.

**Fig. 1:**
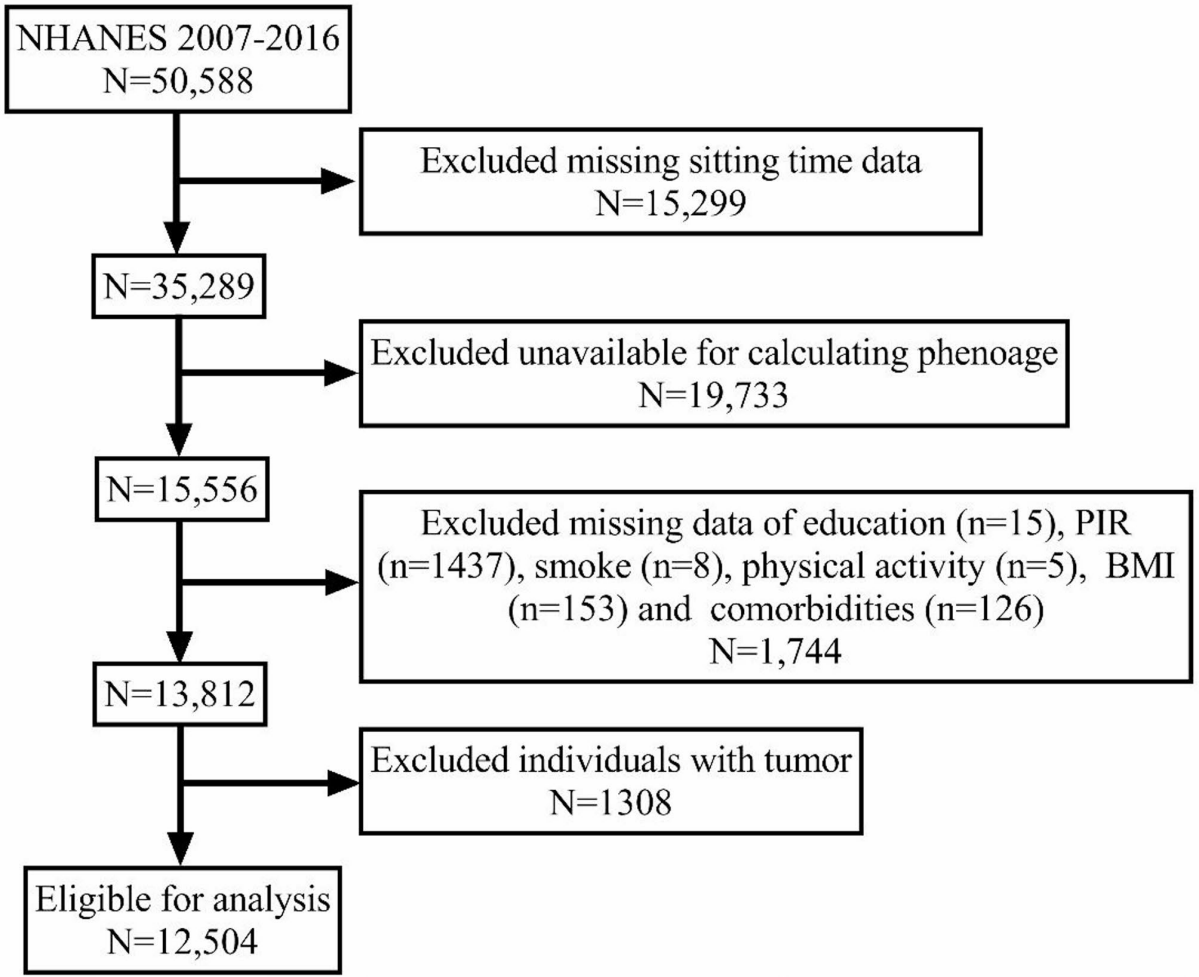
Participant sample flowchar**t**.

The data utilized in the present study were obtained from five successive NHANES cycles, spanning from 2007 to 2016 (*n* = 50,588). We excluded participants with missing data on sitting time (*n* = 15,299), who were unavailable for calculating PhenoAge (*n* = 19,733), with missing data on other covariates (*n* = 1,744), or with tumor (*n* = 1308). Consequently, 12,504 eligible participants aged more than 20 years old were included in the final analytic sample. The complete data integration process was depicted in Fig. 1.

### Outcome measurements

PhenoAge was calculated using chronological age and nine biomarkers: albumin, creatinine, glucose, log [C-reactive protein (CRP)], lymphocyte percentage, mean cell volume, red blood cell distribution width, alkaline phosphatase, and white blood cell count^7,15^. We trained the PhenoAge algorithm with NHANES III data and calculated PhenoAge in NHANES IV cycles from 2007 to 2016 using the “BioAge” R package (https://github.com/dayoonkwon/BioAge)^16^. The final equation for calculating Phenotypic Age is provided below^7,15^:

$$\:Phenotypic\:Age=141.5+\frac{\text{l}\text{n}[-0.00553\times\:\text{l}\text{n}(1-\text{M}\left)\right]}{0.09165}$$

where

$$\:M=1-exp\left(\frac{-1.51714\times\:\text{exp}\left(xb\right)}{0.0076927}\right)$$.

xb = − 19.907 − 0.0336 × albumin + 0.0095 × creatinine + 0.1953 × glucose + 0.0954 × ln(CRP) − 0.0120 × lymphocyte percent + 0.0268 × mean cell volume + 0.3306 × red cell distribution width + 0.00188 × alkaline phosphatase + 0.0554 × white blood cell count + 0.0804 × chronological age.

Besides, phenotypic age acceleration (PhenoAgeAccel) or deceleration/stasis was estimated from the difference between the PhenoAge and chronological age (determined by the date of birth)^17^.

### Exposure measurements

In NHANES, the physical activity questionnaire was based on the Global Physical Activity Questionnaire (GPAQ) which includes questions related to sedentary activities^18^. Daily sitting time was measured by the following question: “On a typical day, how much time do you usually spend sitting at school, at home, getting to and from places, or with friends, including time spent sitting at a desk, traveling in a car or bus, reading, playing cards, watching television, or using a computer?”. Consistent with recent research^19,20^we classified daily sitting time into four categories (< 4, 4–6, 6–8, and ≥ 8 h/day), labeled as Q1, Q2, Q3, and Q4, respectively.

### Covariates

Based on prior researches^21,22^covariates included age, gender (male, female), race (Mexican American, non-Hispanic Black, non-Hispanic White, other Hispanic, and other races), marital status (divorced, married, never married, and others), education level (lower than 12th grade, high school grade, and college grade and above), body mass index (BMI, kg/m^2^) (≤ 20, 20–25, 25–30, and > 30), poverty income ratio (PIR) (< 1.3, 1.3–3.5, and ≥ 3.5), smoke, physical activity (lightly, moderate, and vigorous), self-reported history of diabetes, coronary heart disease (CHD), stroke, congestive heart failure (CHF), and hypertension (HTN). Smoking was defined as having smoked at least 100 cigarettes in life.

### Statistical analyses

Referring to previous NHANES literatures^23,24^we followed NHANES analytical guidelines and applied appropriate sample weights for the enrollment of participants. Continuous variables were expressed as weighted mean ± standard error (SE) and analyzed via the Student’s t-test for normally distributed data or the Mann-Whitney U-test for skewed distribution data, while categorical variables were expressed as weighted percentages and analyzed using the Rao-Scott Chi-square. The baseline characteristics of the participants were compared according to PhenoAge deceleration/stasis and PhenoAgeAccel. Weighted multivariate logistic regression was employed to calculate the odd ratios (OR) and 95% confidence intervals for the association of sitting time and PhenoAge. Three models were constructed: Model 1 was unadjusted, Model 2 was adjusted for age, gender, race, education level, PIR, marital status, and Model 3 incorporated further adjustments for BMI, smoking, physical activity, diabetes, CHD, stroke, CHF, and HTN. Restricted cubic spline regression was used to explore potential non-linear relationships between sitting time and PhenoAge. Referring to previous studies, RCS is particularly suitable for analyzing dose-response relationships with non-linear characteristics^25,26^. Additionally, RCS models with three knots positioned at the (10th, 50th, and 90th) percentiles were applied to further explore the dose-effect relationship. Stratified analyses by potential effect modifiers and tests for multiplicative interactions were performed. Furthermore, we adopted a nonparametric bootstrap method resampled 5000 times to evaluate the indirect impact of sitting time on PhenoAgeAccel mediated through BMI. The proportion mediated was calculated as the ratio of the indirect effect to the total effect, and statistical significance was determined using bootstrapped 95% confidence intervals. This robust method, widely applied in epidemiological studies^27,28^avoids assumptions of normality and provides reliable estimates of mediation effects.

All statistical analyses were performed using R software (version 4.2.2) with a 2-sided *P* < 0.05 considered statistically significant. All regression models were fitted using the svyglm function from the survey package in R.

## Results

### Population characteristics

Table 1 showed the characteristics of the participants. A total of 12,504 eligible participants (weighted population, 100,939,130) with an average age of 45.3 ± 16.0 years were enrolled in the analysis, among whom 48.4% were males. Individuals with PhenoAgeAccel were usually older, more likely to be male, had less education level and longer sitting time, exhibited a higher BMI and lower PIR, more likely to smoke. Additionally, they were more likely to suffer from diabetes, CHD, stroke, CHF, and HTN.

**Table 1:** Baseline characteristics of participants.

Characteristic	Overall	PhenoAge deceleration/stasis	PhenoAgeAccel	*p* value
N, (unweighted)	12,504	9683	2821	
N, (weighted)	100,939,130	81,640,870	19,298,260	
Age (years)	45.3 ± 16.0	44.6 ± 15.7	48.1 ± 16.9	< 0.001
Gender, %				0.008
Female	6439.0 (51.6)	5121.0 (52.3)	1318.0 (48.5)	
Male	6065.0 (48.4)	4562.0 (47.7)	1503.0 (51.5)	
Race, %				< 0.001
Mexican American	2286.0 (8.9)	1812.0 (8.8)	474.0 (9.4)	
Non-Hispanic Black	2388.0 (10.7)	1661.0 (9.4)	727.0 (16.3)	
Non-Hispanic White	5324.0 (67.3)	4221.0 (68.6)	1103.0 (62.0)	
Other Hispanic	1434.0 (5.5)	1103.0 (5.2)	331.0 (6.4)	
Other race	1072.0 (7.6)	886.0 (8.0)	186.0 (5.9)	
Marital status				< 0.001
Divorced	1334.0 (9.7)	950.0 (9.0)	384.0 (12.6)	
Married	6542.0 (56.2)	5219.0 (57.9)	1323.0 (49.0)	
Never married	2262.0 (18.4)	1795.0 (18.5)	467.0 (17.6)	
Others	2366.0 (15.8)	1719.0 (14.6)	647.0 (20.9)	
Education, %				< 0.001
Lower than 12th grade	3327.0 (17.1)	2461.0 (15.8)	866.0 (22.5)	
High school grade	2873.0 (22.7)	2139.0 (21.5)	734.0 (27.5)	
College grade and above	6304.0 (60.3)	5083.0 (62.7)	1221.0 (49.9)	
BMI group, %				< 0.001
≤ 20	542.0 (4.8)	474.0 (5.5)	68.0 (2.2)	
20–25	3040.0 (25.9)	2597.0 (28.3)	443.0 (16.0)	
25–30	4167.0 (33.1)	3433.0 (35.2)	734.0 (24.0)	
> 30	4755.0 (36.2)	3179.0 (31.1)	1576.0 (57.8)	
PIR, %				< 0.001
< 1.3	3987.0 (20.8)	2908.0 (19.0)	1079.0 (28.2)	
1.3–3.5	4838.0 (36.3)	3718.0 (35.7)	1120.0 (38.8)	
≥3.5	3679.0 (42.9)	3057.0 (45.3)	622.0 (33.0)	
Smoke, %	5543.0 (43.8)	3941.0 (40.7)	1602.0 (56.9)	< 0.001
Physical activity, %				0.458
Lightly	7171.0 (53.0)	5509.0 (52.8)	1662.0 (53.8)	
Moderate	2772.0 (24.7)	2181.0 (25.0)	591.0 (23.4)	
Vigorous	2561.0 (22.3)	1993.0 (22.2)	568.0 (22.8)	
Diabetes, %	1367.0 (7.9)	659.0 (4.8)	708.0 (21.2)	< 0.001
CHD, %	422.0 (2.6)	232.0 (1.9)	190.0 (5.7)	< 0.001
Stroke, %	379.0 (2.3)	222.0 (1.8)	157.0 (4.6)	< 0.001
CHF, %	299.0 (1.6)	133.0 (0.9)	166.0 (4.5)	< 0.001
HTN, %	4017.0 (27.9)	2703.0 (24.6)	1314.0 (41.7)	< 0.001
Sedentary activity (hours/day)	5.9 ± 3.4	5.8 ± 3.4	6.3 ± 3.5	< 0.001

### Association of sitting time and phenoageaccel

**Fig. 2:**
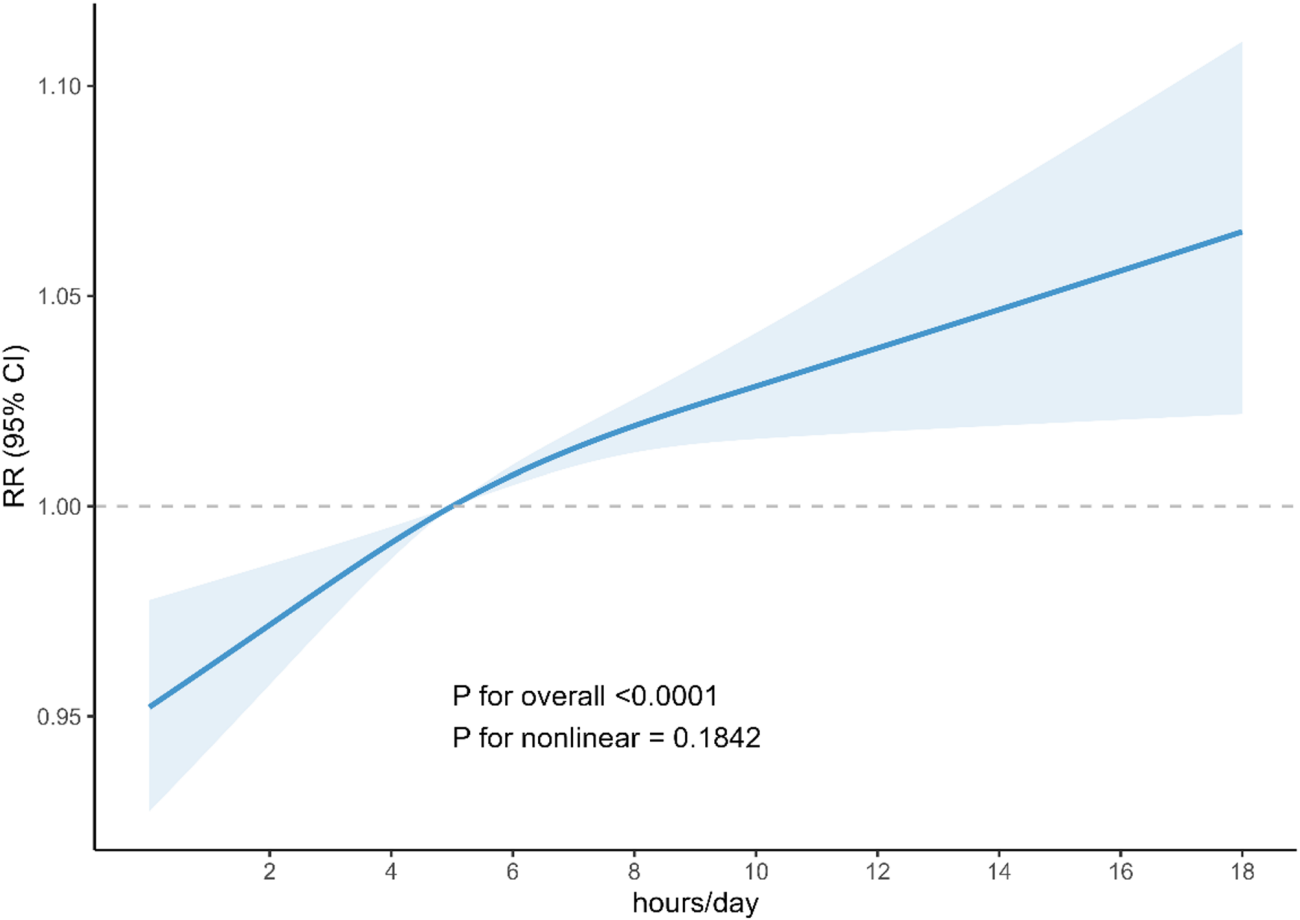
The association of sitting time with PhenoAgeAccel visualized by restricted cubic spline. Risk ratios were adjusted for age, gender, race, education level, PIR, marital status, BMI, smoking, physical activity, diabetes, CHD, stroke, CHF, and HTN.

The results of weighted univariable and multivariable logistic regression models were summarized in Table 2. In crude model (Model 1), adults who sat longer significantly showed greater increases in PhenoAgeAccel (Q2: OR = 1.27, 95%CI = 1.07, 1.51, *p* = 0.007; Q3: OR = 1.28, 95%CI = 1.06, 1.55, *p* = 0.011; Q4: OR = 1.45, 95%CI = 1.25, 1.69, *p* < 0.001) compared to the reference (Q1). Similar association was observed in Model 3. After fully adjusting for age, gender, race, education level, PIR, marital status, BMI, smoking, physical activity, diabetes, CHD, stroke, CHF, and HTN, longer sitting time (Q2: OR = 1.30, 95%CI = 1.06, 1.58, *p* = 0.013; Q3: OR = 1.25, 95%CI = 1.01, 1.55, *p* = 0.038; Q4: OR = 1.58, 95%CI = 1.33, 1.88, *p* < 0.001) statistically significantly corresponded to an increase in PhenoAgeAccel in comparison to the sitting time < 4 h/d. Moreover, Fig. 2 illustrated an approximately positively linear relationship (p for nonlinear = 0.1842) between sitting time and PhenoAgeAccel in a fully adjusted model (Model 3).

**Table 2:** Associations between sitting time and PhenoAgeAccel.

	Model 1			Model 2			Model 3		
	OR	95%CI	p value	OR	95%CI	p value	OR	95%CI	p value
Sitting time									
Q1 (< 4 h/d)	Ref.			Ref.			Ref.		
Q2 (4–6 h/d)	1.27	1.07, 1.51	0.007	1.38	1.16, 1.64	< 0.001	1.30	1.06, 1.58	0.013
Q3 (6–8 h/d)	1.28	1.06, 1.55	0.011	1.43	1.17, 1.74	< 0.001	1.25	1.01, 1.55	0.038
Q4 (≥ 8 h/d)	1.45	1.25, 1.69	< 0.001	1.82	1.54, 2.15	< 0.001	1.58	1.33, 1.88	< 0.001
P for trend			< 0.001			< 0.001			< 0.001

Model 1 was unadjusted.

Model 2 was adjusted for age, gender, race, education level, PIR, and marital status.

Model 3 was adjusted for age, gender, race, education level, PIR, marital status, BMI, smoking, physical activity, diabetes, CHD, stroke, CHF, and HTN.

### Stratified analyses

**Fig. 3:**
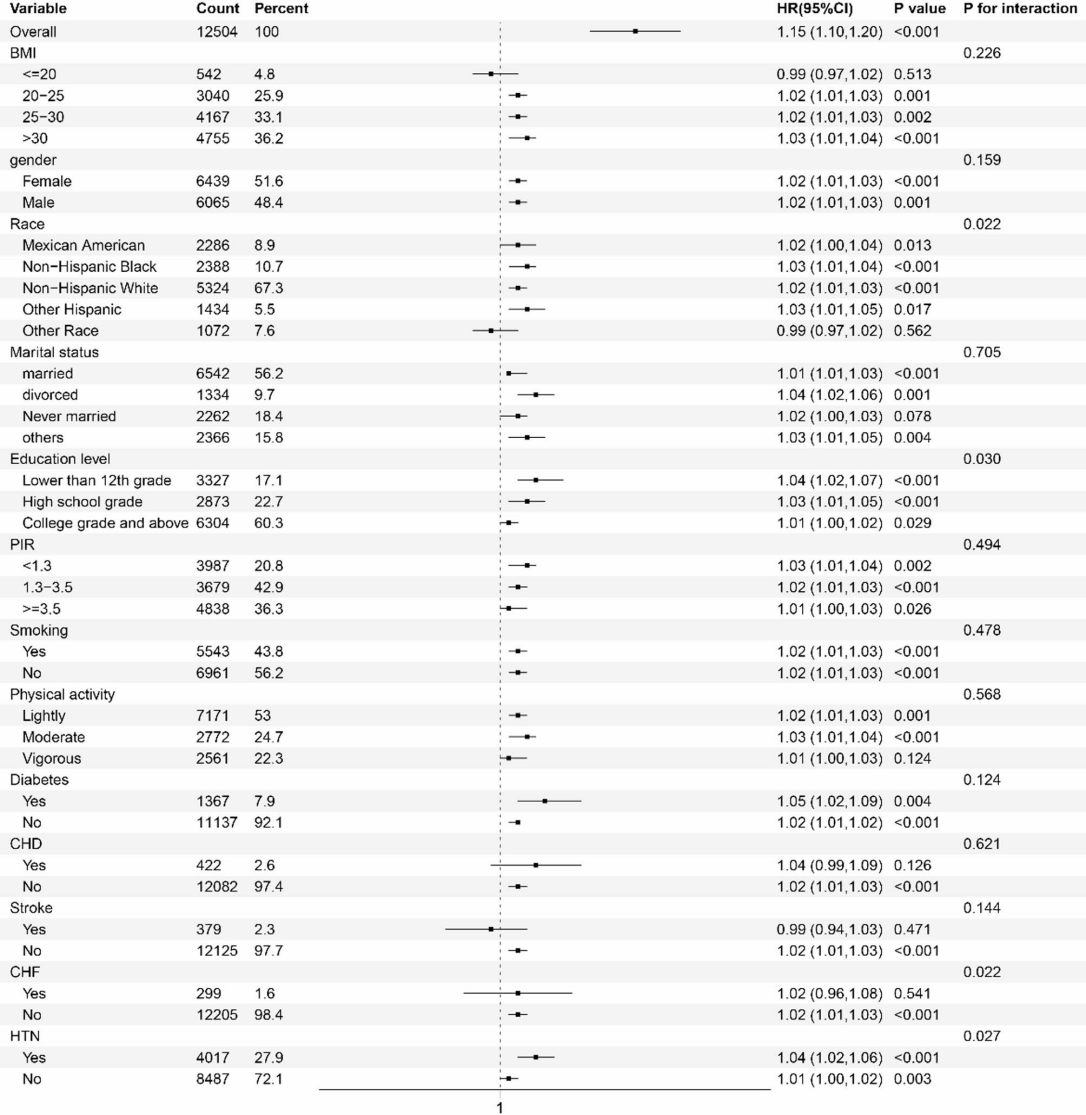
Subgroup analysis for the association between sitting time and PhenoAgeAccel. HRs were adjusted for age, gender, race, education level, PIR, marital status, BMI, smoking, physical activity, diabetes, CHD, stroke, CHF, and HTN.

We conducted stratified analyses to explored the relationship between sitting time and PhenoAgeAccel based on BMI, gender, race, marital status, education, PIR, smoking, physical activity, diabetes, CHD, stroke, CHF, and HTN (Fig. 3). The findings indicated that race, education level, CHF and HNT could serve as moderating factors in the relationship between sitting time and PhenoAgeAccel (P for interaction < 0.05).

### Mediation analyses

**Fig. 4:**
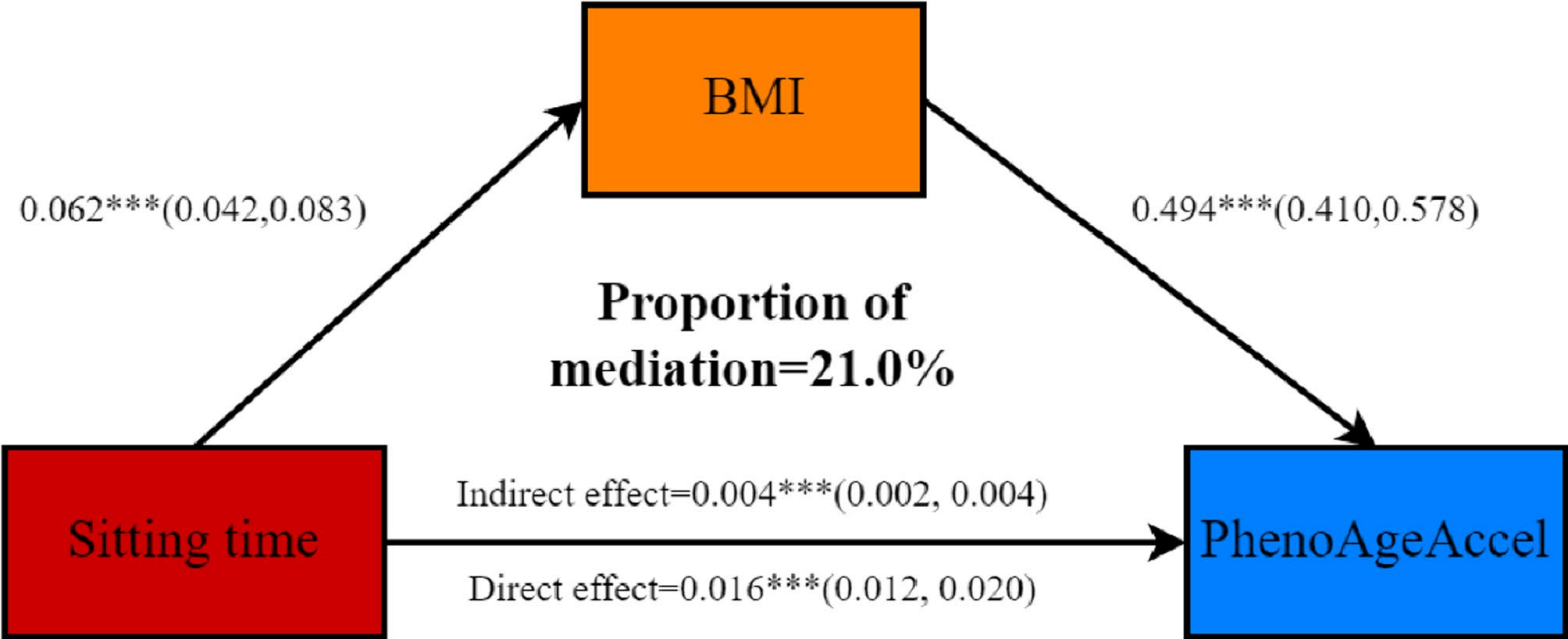
The mediating effect of BMI on the relationship between sitting time and PhenoAgeAccel. Adjusted for age, gender, race, education level, PIR, marital status, BMI, smoking, physical activity, diabetes, CHD, stroke, CHF, and HTN.

Mediation analyses revealed the mediating effect of BMI on the correlation between sitting time and PhenoAgeAccel. The indirect mediated effect through BMI was 0.004 (95%CI: 0.002–0.004). The results indicated that BMI mediated 21.0% variance of the total effect and was a significant mediator of the association between sitting time and PhenoAgeAccel. Figure 4 displayed the mediation pathway model.

## Discussion

Using the 12,504 nationally representative U.S. adult participants in the NHANES, we found a strong positive correlation between sitting time and PhenoAgeAccel. The dose–response analysis showed a linear association of sitting time and PhenoAgeAccel and the mediation analyses suggested the role of BMI as a mediator in this relationship.

With the changes in lifestyle attributed to technological advances, sitting time was prolonged and the negative health effects of sedentary behaviors attracted attention. In 2023, You et al.^11^ enrolled 6,439 representative adults from NHANES to conduct a cross-sectional study and concluded that prolonged sedentary time was associated with elevated PhenoAge. Lately, Lu et al., using data from the Guangzhou Biobank Cohort Study, triangulate observational and mendelian randomization studies and find that sedentary behavior had a detrimental impact on PhenoAgeAccel^12^. These results were consistent with ours. Nevertheless, our study adjusted for more confounding variables, explored the dose-response relationship between sitting time and PhenoAgeAccel, and the role of BMI in this relationship.

There were various potential pathways through which sedentary behavior might affect aging. A randomized controlled trial found a negative correlation between sitting time and telomere length^29^. However, it should be noted that only 49 older people were included in the trial. Hence, the results could simply be a chance finding due to the inadequate sample size^30^. Moreover, it had been investigated that nutrient sensing pathways could modulate longevity^31^. Meanwhile, sedentary behavior was able to blunt the nutrient signaling pathways responsible for muscle growth such as insulin/insulin-like growth factor 1 (IGF-1) signaling (IIS) pathways, mechanistic target of rapamycin (mTOR), growth hormone (GH), and testosterone signaling. Furthermore, sedentary behavior was possibly associated with genomic instability, epigenetic alterations, loss of proteostasis, mitochondrial dysfunction, which aggravates the aging process^32^.

In our mediation analysis, we found that sedentary behavior accelerated aging in part by increasing BMI, which suggested a potential underlying mechanism. Consistent with previous research, sedentary behavior, characterized by low muscle activity, high energy intake, and reduced physical exercise, was related with higher BMI^33–37^. This increased BMI could induce a highly inflammatory systemic milieu with perturbations of white blood cell counts^38,39^. An observational twin study conducted by Sara et al.^40^ demonstrated a significant association between BMI and accelerated epigenetic aging, independent of genetic factors, potentially mediated by insulin resistance. Furthermore, Sangmi et al.^41^ found that BMI might accelerate aging by shortening telomere length. Moreover, aging of the immune system, reactive oxygen species and redox homeostasis were also confirmed to potentially play roles in obesity-induced aging^13^.

Subgroup analyses revealed significant effect modifications by race, education level, CHF, and HNT status. The association between sedentary time and PhenoAgeAccel was particularly pronounced in Non-Hispanic Black individuals, those with low education levels, those with HTN, and those without CHF, suggesting potential socioeconomic and cardiometabolic disparities in susceptibility to sedentary-related aging. While these findings underscore high-risk populations for prioritized intervention, residual confounding and small subgroup samples warrant cautious interpretation. Prospective studies with repeated measures are needed to verify these interactions.

It should be acknowledged that this study inevitably had several shortcomings. First, in our research, daily sitting time was assessed by the self-report, which may introduce recall bias. Second, the cross-sectional study design was unavailable to determine causality, which require prospective studies to ascertain. Third, some baseline information, including sitting time and history of diseases, was based on recollection and self-report, which might lead to recall bias. Fourth, although we had tried to take into account as many variables as possible, there were still some unaccounted variables (like energy intake, occupation, and psychological factors) that could influence the relationship between sitting time and aging. Fifth, we didn’t further distinguish work sitting time and leisure sitting time, so we failed to separately explore the impacts of these contexts in these PhenoAgeAccel. Finally, while our stringent inclusion criteria ensured data quality for the mediation analysis, only 24% (12,504/50,588) of the original NHANES participants met all eligibility requirements. The limited sample size may affect the generalizability of our findings to populations with different characteristics .

## Conclusion

Using a representative nationwide population, the study determined the association between sitting time and emerging PhenoAge index. Our findings revealed a strong significant and independent relationship between sedentary behavior and PhenoAgeAccel, which exhibited a proximate linear dose-response relationship. Furthermore, BMI served as a mediator of the correlation between sitting time and PhenoAgeAccel.

## Data Availability

All data analyzed during the current study are publicly available on the NHANES website (https://www.cdc.gov/nchs/nhanes/index.htm).

## Abbreviations

BMI: Body mass index
CDC: Centers for Disease Control and Prevention
CHD: Coronary heart disease
CHF: Congestive heart failure
CI: Confidence interval
CRP: C-reactive protein
GPAQ: Global Physical Activity Questionnaire
HTN: Hypertension
NCHS: National Center for Health Statistics
NHANES: National Health and Nutrition Examination Survey
OR: Odd ratio
PhenoAge: Phenotypic age
PhenoAgeAccel: Phenotypic age acceleration
PIR: Poverty income ratio
WHO: World Health Organization

## References

[1] Tremblay, M. S. et al. Sedentary behavior research network (SBRN) – Terminology consensus project process and outcome. *Int. J. Behav. Nutr. Phys. Activity*. **14**(1). 10.1186/s12966-017-0525-8 (2017). 10.1186/s12966-017-0525-8 PMC5466781 28599680

[2] Li, S. et al. Association of sitting time with mortality and cardiovascular events in high-income, middle-income, and low-income. *JAMA Cardiol.* **7**(8), 796–807. 10.1001/jamacardio.2022.1581 (2022). 35704349 10.1001/jamacardio.2022.1581 PMC9201743

[3] Liu, M. et al. Association of accelerometer-measured physical activity intensity, sedentary time, and exercise time with incident Parkinson’s disease. *NPJ Digit. Med.* **6**(1), 224. 10.1038/s41746-023-00969-7 (2023). 38017114 10.1038/s41746-023-00969-7 PMC10684568

[4] Lin, Z. et al. Correlation between sedentary activity, physical activity and bone mineral density and fat in america: National health and nutrition examination survey, 2011–2018. *Sci. Rep.* **13**(1), 10054. 10.1038/s41598-023-35742-z (2023). 37344579 10.1038/s41598-023-35742-z PMC10284806

[5] Bankoski, A. et al. Sedentary activity associated with metabolic syndrome independent of physical activity. *Diabetes Care*. **34**(2), 497–503. 10.2337/dc10-0987 (2011). 21270206 10.2337/dc10-0987 PMC3024375

[6] Bautmans, I. et al. WHO working definition of vitality capacity for healthy longevity monitoring. *Lancet Healthy Longev.* **3**(11), e789–e796. 10.1016/S2666-7568(22)00200-8 (2022). 36356628 10.1016/S2666-7568(22)00200-8 PMC9640935

[7] Basu, S. et al. A new aging measure captures morbidity and mortality risk across diverse subpopulations from NHANES IV: A cohort study. *PLoS Med.* **15**(12). 10.1371/journal.pmed.1002718 (2018). 10.1371/journal.pmed.1002718 PMC6312200 30596641

[8] Thomas, A., Belsky Daniel, W., Gu, Y. & Duque, G. Healthy lifestyle behaviors and biological aging in the U.S. National health and nutrition examination surveys 1999–2018. *Journals Gerontology: Ser. A*. **78**(9), 1535–1542. 10.1093/gerona/glad082 (2023). 10.1093/gerona/glad082 PMC10460553 36896965

[9] You, Y. et al. Accelerometer-measured physical activity patterns are associated with phenotypic age: isotemporal substitution effects. *Heliyon* **9**(9). 10.1016/j.heliyon.2023.e19158 (2023). 10.1016/j.heliyon.2023.e19158 PMC10558316 37810111

[10] Cao, X. et al. Weight change across adulthood and accelerated biological aging in middle-aged and older adults. *Am. J. Clin. Nutr.* **117**(1), 1–11. 10.1016/j.ajcnut.2022.10.020 (2023). 36789928 10.1016/j.ajcnut.2022.10.020

[11] You, Y. et al. Accelerometer-measured physical activity patterns are associated with phenotypic age: isotemporal substitution effects. *Heliyon* **9**(9), e19158. 10.1016/j.heliyon.2023.e19158 (2023). 37810111 10.1016/j.heliyon.2023.e19158 PMC10558316

[12] Lu, T. Y. et al. Active longevity and aging: dissecting the impacts of physical and sedentary behaviors on longevity and age acceleration. *GeroScience*10.1007/s11357-024-01329-3 (2024). 39230773 10.1007/s11357-024-01329-3 PMC12181555

[13] Tam, B. T., Morais, J. A. & Santosa, S. Obesity and ageing: two sides of the same coin. *Obes. Rev*. **21**(4), e12991. 10.1111/obr.12991 (2020). 32020741 10.1111/obr.12991

[14] Zipf, G. et al. National health and nutrition examination survey: plan and operations, 1999–2010. *Vital Health Stat. 1*(56) (2013). 25078429

[15] Wu, Z. et al. Association of physical activity with phenotypic age among populations with different breakfast habits. *Nutrients* **16**(5). 10.3390/nu16050575 (2024). 10.3390/nu16050575 PMC10934488 38474704

[16] Liu, W. et al. Oxidative stress factors mediate the association between life’s essential 8 and accelerated phenotypic aging: NHANES 2005–2018. *J. Gerontol. Ser. A*. **79**(1). 10.1093/gerona/glad240 (2024). 10.1093/gerona/glad240 37813096

[17] Lê, B. M. et al. Characterizing epigenetic aging in an adult sickle cell disease cohort. *Blood Adv.* **8**(1), 47–55. 10.1182/bloodadvances.2023011188 (2024). 37967379 10.1182/bloodadvances.2023011188 PMC10784677

[18] Keating, X. D. et al. Reliability and concurrent validity of global physical activity questionnaire (GPAQ): A systematic review. *Int J. Environ. Res. Public. Health Oct.* **26**(21). 10.3390/ijerph16214128 (2019). 10.3390/ijerph16214128 PMC6862218 31717742

[19] Zhou, H. et al. Association of daily sitting time and coffee consumption with the risk of all-cause and cardiovascular disease mortality among US adults. *BMC Public. Health*. **24**(1). 10.1186/s12889-024-18515-9 (2024). 10.1186/s12889-024-18515-9 PMC11022421 38632571

[20] Li, Y. et al. Association between daily sitting time and kidney stones based on the National health and nutrition examination survey (NHANES) 2007–2016: A cross-sectional study. *Int. J. Surg.*10.1097/js9.0000000000001560 (2024). 38768465 10.1097/JS9.0000000000001560 PMC11325893

[21] You, Y. et al. Inverted U-shaped relationship between sleep duration and phenotypic age in US adults: a population-based study. *Sci. Rep.* **15**(1), 6247. 10.1038/s41598-024-56316-7 (2024). 10.1038/s41598-024-56316-7 PMC10940593 38486063

[22] You, Y. et al. Dose-response relationship between leisure-time physical activity and metabolic syndrome in short sleep US adults: evidence from a nationwide investigation. *Appl Physiol. Nutr. Metab.*. **50**, 1–10. 10.1139/apnm-2024-0347 (2025). 39993280 10.1139/apnm-2024-0347

[23] You, Y. et al. Exploring the potential relationship between short sleep risks and cognitive function from the perspective of inflammatory biomarkers and cellular pathways: insights from population-based and mice studies. *CNS Neurosci. Ther.*. **30**(5), e14783. 10.1111/cns.14783 (2024). 38797980 10.1111/cns.14783 PMC11128714

[24] You, Y., Mo, L., Tong, J., Chen, X. & You, Y. The role of education attainment on 24-hour movement behavior in emerging adults: evidence from a population-based study. *Front. Public. Health*. **12**, 1197150. 10.3389/fpubh.2024.1197150 (2024). 38292911 10.3389/fpubh.2024.1197150 PMC10824836

[25] You, Y. Accelerometer-measured physical activity and sedentary behaviour are associated with C-reactive protein in US adults who get insufficient sleep: A threshold and isotemporal substitution effect analysis. *J. Sports Sci.* **42**(6), 527–536. 10.1080/02640414.2024.2348906 (2024). 38695324 10.1080/02640414.2024.2348906

[26] You, Y. et al. Saturation effects of the relationship between physical exercise and systemic immune inflammation index in the short-sleep population: a cross-sectional study. *BMC Public. Health*. **17**(1), 1920. 10.1186/s12889-024-19432-7 (2024). 10.1186/s12889-024-19432-7 PMC11256404 39020383

[27] You, Y. et al. The role of dietary intake of live microbes in the association between leisure-time physical activity and depressive symptoms: a population-based study. *Appl Physiol. Nutr. Metab*. **1**(8), 1014–1024. 10.1139/apnm-2023-0550 (2024). 10.1139/apnm-2023-0550 38569203

[28] You, Y. et al. Mediation role of recreational physical activity in the relationship between the dietary intake of live microbes and the systemic Immune-Inflammation index: A real-world cross-sectional study. *Nutrients*. **8**(6). 10.3390/nu16060777 (2024). 10.3390/nu16060777 PMC10974920 38542688

[29] Sjögren, P. et al. Stand up for health–avoiding sedentary behaviour might lengthen your telomeres: secondary outcomes from a physical activity RCT in older people. *Br. J. Sports Med.* **48**(19), 1407–1409. 10.1136/bjsports-2013-093342 (2014). 25185586 10.1136/bjsports-2013-093342

[30] Haldar, P., Ramesh, V. & Kant, S. Effect of sedentary activity on telomere length may not be so straightforward. *Br. J. Sports Med.* **49**(24), 1604–1604. 10.1136/bjsports-2014-094473 (2015). 25573617 10.1136/bjsports-2014-094473

[31] Barzilai, N., Huffman, D. M., Muzumdar, R. H. & Bartke, A. The critical role of metabolic pathways in aging. *Diabetes* **61**(6), 1315–1322. 10.2337/db11-1300 (2012). 22618766 10.2337/db11-1300 PMC3357299

[32] Raffin, J. et al. Sedentary behavior and the biological hallmarks of aging. *Ageing Res. Rev.* **83**10.1016/j.arr.2022.101807 (2023). 10.1016/j.arr.2022.101807 36423885

[33] Hu, F. B., Li, T. Y., Colditz, G. A., Willett, W. C. & Manson, J. E. Television watching and other sedentary behaviors in relation to risk of obesity and type 2 diabetes mellitus in women. *JAMA* **289**(14), 1785–1791 (2003). 12684356 10.1001/jama.289.14.1785

[34] Du, H. et al. Physical activity and sedentary leisure time and their associations with BMI, waist circumference, and percentage body fat in 0.5 million adults: the China kadoorie biobank study. *Am. J. Clin. Nutr.* **97**(3), 487–496. 10.3945/ajcn.112.046854 (2013). 23364014 10.3945/ajcn.112.046854 PMC4345799

[35] Mummery, W. K., Schofield, G. M., Steele, R., Eakin, E. G. & Brown, W. J. Occupational sitting time and overweight and obesity in Australian workers. *Am. J. Prev. Med.* **29**(2), 91–97 (2005). 16005804 10.1016/j.amepre.2005.04.003

[36] Mozaffarian, D., Hao, T., Rimm, E. B., Willett, W. C. & Hu, F. B. Changes in diet and lifestyle and long-term weight gain in women and men. *N Engl. J. Med.* **364**(25), 2392–2404. 10.1056/NEJMoa1014296 (2011). 21696306 10.1056/NEJMoa1014296 PMC3151731

[37] Heinonen, I. et al. Sedentary behaviours and obesity in adults: the cardiovascular risk in young Finns study. *BMJ Open*. **20**(6). 10.1136/bmjopen-2013-002901 (2013). 10.1136/bmjopen-2013-002901 PMC3669715 23794543

[38] Panagiotakos, D. B., Pitsavos, C., Yannakoulia, M., Chrysohoou, C. & Stefanadis, C. The implication of obesity and central fat on markers of chronic inflammation: the ATTICA study. *Atherosclerosis*. **183**(2), 308–315. 10.1016/j.atherosclerosis.2005.03.010 (2005). 16285994 10.1016/j.atherosclerosis.2005.03.010

[39] Visser, M., Bouter, L. M., McQuillan, G. M., Wener, M. H. & Harris, T. B. Low-grade systemic inflammation in overweight children. *Pediatrics*. **107**(1), E13. 10.1542/peds.107.1.e13 (2001). 11134477 10.1542/peds.107.1.e13

[40] Lundgren, S. et al. BMI is positively associated with accelerated epigenetic aging in twin pairs discordant for body mass index. *J Intern. Med.* **292**(4), 627–640. 10.1111/joim.13528 (2022). 35699258 10.1111/joim.13528 PMC9540898

[41] Kim, S. et al. Obesity and weight gain in adulthood and telomere length. *Cancer Epidemiol. Biomarkers Prev.* **18**(3), 816–820. 10.1158/1055-9965.EPI-08-0935 (2009). 19273484 10.1158/1055-9965.EPI-08-0935 PMC2805851

